# Prevalence of *Rickettsiales* in ticks removed from the skin of outdoor workers in North Carolina

**DOI:** 10.1186/s13071-014-0607-2

**Published:** 2014-12-23

**Authors:** Sangmi Lee, Madhavi L Kakumanu, Loganathan Ponnusamy, Meagan Vaughn, Sheana Funkhouser, Haley Thornton, Steven R Meshnick, Charles S Apperson

**Affiliations:** Department of Entomology, North Carolina State University, Campus Box 7647, Raleigh, NC 27695-7647 USA; Department of Epidemiology, Gillings School of Global Public Health, University of North Carolina, Chapel Hill, NC 27599 USA; Present address: National Institutes of Health, Bethesda, MD 20892 USA

**Keywords:** Ticks, *Rickettsiales* pathogens, *Rickettsia*, *Ehrlichia*, Reverse line blot hybridization

## Abstract

**Background:**

Tick-transmitted rickettsial diseases, such as ehrlichiosis and spotted fever rickettsiosis, are significant sources of morbidity and mortality in the southern United States. Because of their exposure in tick-infested woodlands, outdoor workers experience an increased risk of infection with tick-borne pathogens. As part of a double blind randomized-controlled field trial of the effectiveness of permethrin-treated clothing in preventing tick bites, we identified tick species removed from the skin of outdoor workers in North Carolina and tested the ticks for *Rickettsiales* pathogens.

**Methods:**

Ticks submitted by study participants from April-September 2011 and 2012 were identified to species and life stage, and preliminarily screened for the genus *Rickettsia* by nested PCR targeting the 17-kDa protein gene. *Rickettsia* were further identified to species by PCR amplification of 23S-5S intergenic spacer (IGS) fragments combined with reverse line blot hybridization with species-specific probes and through cloning and nucleotide sequence analysis of 23S-5S amplicons. Ticks were examined for *Ehrlichia* and *Anaplasma* by nested PCR directed at the *gltA,* antigen-expressing gene containing a variable number of tandem repeats, 16S rRNA, and *groESL* genes.

**Results:**

The lone star tick (*Amblyomma americanum*) accounted for 95.0 and 92.9% of ticks submitted in 2011 (*n* = 423) and 2012 (*n* = 451), respectively. Specimens of American dog tick (*Dermacentor variabilis*), Gulf Coast tick (*Amblyomma maculatum*) and black-legged tick (*Ixodes scapularis*) were also identified. In both years of our study, 60.9% of ticks tested positive for 17-kDa. “*Candidatus* Rickettsia amblyommii”, identified in all four tick species, accounted for 90.2% (416/461) of the 23S-5S-positive samples and 52.9% (416/787) of all samples tested. Nucleotide sequence analysis of *Rickettsia*-specific 23S-5S IGS, *ompA* and *gltA* gene fragments indicated that ticks, principally *A. americanum*, contained novel species of *Rickettsia*. Other *Rickettsiales*, including *Ehrlichia ewingii*, *E. chaffeensis*, *Ehrlichia* sp. (Panola Mountain), and *Anaplasma phagocytophilum*, were infrequently identified, principally in *A. americanum*.

**Conclusions:**

We conclude that in North Carolina, the most common rickettsial exposure is to *R. amblyommii* carried by *A. americanum*. Other *Rickettsiales* bacteria, including novel species of *Rickettsia*, were less frequently detected in *A. americanum* but are relevant to public health nevertheless.

**Electronic supplementary material:**

The online version of this article (doi:10.1186/s13071-014-0607-2) contains supplementary material, which is available to authorized users.

## Background

Tick-transmitted rickettsial diseases are a significant source of morbidity and mortality globally [1,2]. In the United States, spotted fever rickettsioses (SFR) and ehrlichioses are frequently reported causes of tick-borne rickettsial illnesses [3]. Rocky Mountain spotted fever (RMSF) is the most significant SFR, because it is a potentially fatal disease [1]. Cases of SFR (including RMSF) have escalated in recent years [4]. In 2011, a larger number of cases were reported to the CDC than in any year since 1920 [3]. Of 43 states reporting SFR cases, six states in the mid-Atlantic and south central regions (Arkansas, Missouri, North Carolina, Oklahoma, Tennessee, and Virginia) reported 70% of the 2,802 cases reported [3].

The risk of tick-transmitted diseases is an important concern of forestry, military personnel and other professionals engaged in outdoor work, because of their exposure in tick-infested woodlands [5-7]. Avoidance of tick-transmitted illness can be achieved through the use of personal protection methods against tick bites, such as application of chemical repellents to clothing and exposed skin [8]. Because of its persistence and fast action, the synthetic pyrethroid acaricide permethrin is an effective tick repellent when impregnated into clothing [8-10]. Recently permethrin-impregnated clothing was shown to effectively prevent tick bites in a double blind randomized-controlled field trial involving a cohort of outdoor workers in North Carolina [11]. In this study, participants self-reported tick bites and collected ticks from their skin. Here we present a comparative evaluation of the tick species biting study participants and results of molecular analyses of the ticks for selected *Rickettsiales* bacterial pathogens.

## Methods

### Collection and processing of ticks

Ticks analyzed were collected from the skin of outdoor workers who participated in an evaluation of the effectiveness of clothing impregnated with the repellent/insecticide permethrin in preventing tick bites [11]. Study participants were employed by the North Carolina Division of Forest Resources, the North Carolina Division of Parks and Recreation or the North Carolina Wildlife Resources Commission and worked in central and eastern North Carolina. Ticks, collected by each participant from April to September in 2011 and 2012, were stored in separate vials containing the DNA preservative propylene glycol [12]. Yields of DNA from lone star tick adults preserved in propylene glycol were comparable to ticks preserved in ethanol (unpublished data). Each month, vials were mailed to North Carolina State University, where ticks were processed. Specimens were enumerated by life stage for each species, after which they were stored in ethanol at −80°C until DNA was extracted. Tick nymphs and adults were stored individually but some larvae were grouped into pools of up to five specimens. In 2011, 53 larvae were tested after they were pooled (9 and 1 pools containing five and three larvae, respectively, and 5 individual larvae). In 2012, 64 larvae were tested (11, 2 and 1 pools containing five, three and two larvae, respectively, and one individual larva). DNA was extracted from these pools as described below.

### Extraction of DNA from tick samples

Genomic DNA was extracted from individual adults, nymphs and pools of larvae using methods previously described [13]. Crude DNA samples were purified with the Wizard DNA Clean-Up System (Promega, Madison, WI, USA) and the purified DNA was quantified with a NanoDrop (Thermo Scientific, Wilmington, DE, USA). DNA samples were then stored at −80°C for later use.

### Molecular detection of *Rickettsiales* pathogens

Including DNA extractions from pools of larvae, 787 tick DNA samples (385 and 402 samples in 2011 and 2012, respectively) were prepared and analyzed for pathogens. Genomic DNA was used as template in PCR assays to amplify fragments of gene targets specific for the genus *Rickettsia*, *Ehrlichia chaffeensis*, *E. ewingii* and *Anaplasma phagocytophilum*. The oligonucleotide primers used are shown in Additional file 1: Table S1 and were synthesized by Integrated DNA Technologies (Coralville, IA, USA). Primary amplification of nested PCR reactions was conducted in a 10 μl reaction mixture composed of 1 μl of genomic DNA, 0.5 μl of forward primer (10 μM), 0.5 μl of reverse primer (10 μM), 5 μl of 2× AmpliTaq Gold PCR Master Mix (Applied Biosystems, Grand Island, NY, USA) and 3 μl of nuclease-free water (QIAGEN, Valencia, CA, USA). When pooled samples of tick DNA extracts were used (see explanation below), the reaction volume was increased to 20 μl and 5 μl of the pooled DNA sample was used as template. The nested PCR reaction mix consisted of 1 μl of the first PCR product as template DNA, 1 μl of forward primer (10 μM), 1 μl of reverse primer (10 μM), 10 μl of 2× AmpliTaq Gold PCR Master Mix and 7 μl of nuclease-free water. Quality control measures for PCR assays included negative controls (no template DNA) and positive controls (employing DNA from known species of *Rickettsia* as template) that were amplified in parallel with tick samples in each PCR run. Tick processing was carried out in a non-ventilated PCR enclosure. PCR reactions were prepared in the PCR enclosure or a laminar flow hood. Before they were used, these work areas were thoroughly cleaned with ethanol and exposed to UV light. The PCR conditions for individual rickettsial groups are discussed below.

### Genus *Rickettsia*

All the tick samples were first screened for genus *Rickettsia* with primers targeting the *Rickettsia* genus-specific 17-kDa protein gene (Additional file 1: Table S1) [14]. Primary amplification was performed with primers 17kD1_F, 17kD1_R using a thermocycler program consisting of an initial denaturation of 95°C for 10 min; 35 cycles of denaturation at 95°C for 30 s, annealing at 47°C for 30 s, and extension at 72°C for 1 min; and final extension of 72°C for 10 min. Primers 17kN1_F and 17kN2_R were used in the nested amplification. The thermocycler conditions were similar to the initial PCR amplification except that the annealing temperature was increased to 50°C and the cycles were repeated 30 times. To visualize the nested PCR amplicon, 3 μl of each PCR product was electrophoresed in a 1.2% agarose gel containing ethidium bromide in 0.5× TAE buffer. Subsequently, 17-kDa-positive samples were further examined with a reverse-line blot (RLB) hybridization assay as described below to identify *Rickettsia* to species.

### Development of a PCR-RLB hybridization assay

A PCR-RLB hybridization assay was developed to differentiate 10 *Rickettsia* species (seven spotted fever group [SFG] species, one typhus group [TG] species and, *R. bellii* and *R. canadensis*, representing two ancestral group species), which included confirmed human pathogens (*R. conorii*, *R. rickettsii*, *R. parkeri*, and *R. typhi*) [15] and a *Rickettsia* species (“*Candidatus* R. amblyommii”) that has been frequently detected in North Carolina ticks [16-18]. Control DNAs for *Rickettsia* species were obtained from the Rickettisal Zoonoses Branch, the Centers for Disease Control and Prevention (Atlanta, GA, USA). *R. conorii* and *R. typhi* were included in our study because of their availability, not because we expected these rickettsiae to be detected in the ticks tested.

The assay combined PCR amplification of a ~400 bp fragment of the variable 23S-5S IGS and RLB hybridization procedures as previously published [19,20]. Briefly, PCR was conducted using the parameters described by Jado *et al*. [19] in a 20 μl reaction mixture consisting of 1 μl of tick DNA extraction, 1 μl of primer RCK/23-5-F (10 μM), 1 μl of primer RCK/23-5-F (10 μM), 10 μl of AmpliTaq Gold PCR Master Mix, and 7 μl of nuclease-free water (Additional file 1: Table S2) [19]. The primers used in this reaction were biotin-modified at the 5′ end and were purchased from Invitrogen (Grand Island, NY, USA). One microliter of PCR products was visualized in a 1.2% agarose gel, containing ethidium bromide. Those samples displaying a band were diluted by mixing 10 μl of the PCR product and 180 μl of 2× SSPE/0.1% SDS solution, and used in RLB hybridization assays described below.

Probes used in the RLB hybridization are shown in Additional file 1: Table S2. Out of 16 probes, seven were previously published by Jado *et al*. [19], and the remaining nine were designed for our study. In designing probes, we retrieved DNA sequences of different *Rickettsia* species from the National Center for Biotechnology Information (NCBI, http://www.ncbi.nlm.nih.gov/) aligned them with ClustalW2 (http://www.ebi.ac.uk/Tools/msa/clustalw2/) [21] with the region between primers RCK/23-5-F and RCK/23-5-R. Short nucleotide sequences specific to particular *Rickettsia* species were identified through visual inspection and used as probes in the hybridization. All oligonucleotide probes were synthesized with 5′-terminal amino group label by Invitrogen. Probes were dissolved in nuclease-free water to 100 μM and further diluted to 200 μl at 1 and 2 μM with 0.5 M NaHCO_3_ (pH 8.4). For probes whose signal was consistently weak (P-MON3, P-MON4, P-RHIPI and P-RIC), higher concentration dilutions (4 and 8 μM) were also prepared.

The probes were hybridized with PCR products as described previously by Kong and Gilbert [20] with modifications in their protocol. The RLB hybridization temperature was lowered to 52°C from 55-60°C and stripping solution concentration and temperature were modified to 0.5% SDS and 60°C instead of 1% SDS and 80°C, respectively. Bound PCR products were detected by chemiluminescence with a ChemiDot-It^TS2^ Imaging System (UVP, Upland, CA, USA) following incubation of the membrane in ECL detection liquid (Amersham, Little Chalfont, Buckinghamshire, United Kingdom). Some cross-hybridization was anticipated because the amino-labeled probes were 17–25 bp in size with some of the probe sequences varying by a single or just a few nucleotides. Consequently, specificity of the probes and optimal RLB hybridization conditions were determined by RLB hybridizations of the probes with control DNAs representing the 10 target *Rickettsia* species. This RLB hybridization assay was applied to all tick DNA samples that were positive for genus *Rickettsia* in the 17-kDa nested PCR assay and generated a ~400 bp amplicon in the PCR targeting the 23S-5S IGS. In all the RLB hybridizations, control DNA extractions were also included for quality control of the assay.

### Cloning and sequence analysis of *Rickettsia* spp.

We amplified, cloned and sequenced 23S-5S gene fragments to identify *Rickettsia* spp. or to verify RLB assay results. Amplicons from some ticks failed to hybridize to any probe (further referred to as unknowns). Generally, these amplicons were detected as faint gel bands in the aforementioned PCR targeting 23S-5S IGS. All the unknown samples were amplified again using a larger amount (2 μl) of genomic DNA. Fifteen samples produced visible gel bands of the appropriate size, which were excised, and the DNA was extracted and gel purified using the QIAquick gel extraction kit (catalog no. 28704, QIAGEN). Also, the 23S-5S amplicons of 24 samples hybridized to probes for two *Rickettsia* species (further referred as co-infected samples). All 24 samples were amplified again with 2 μl of genomic DNA and 17 samples that produced bright bands of the correct size were excised and purified. Additionally, DNA samples from three ticks identified to be infected with either *R. parkeri* or *R. montanensis* were amplified again with 2 μl genomic DNA, cloned and sequenced. In summary, a total of 35 samples (15 unknowns, 17 co-infected and 3 samples that were identified as *R*. *parkeri*/ *R. montanenis*) were cloned and sequenced. To generate a 23S-5S rRNA clone library for these three sets of samples, purified DNA for each amplicon was inserted into the plasmid vector pGEM-T (Cat. No A3610, pGEM–T Vector System II; Promega) as specified by the manufacturer. White colonies were picked and checked for the presence of the insert by amplifying the clones with universal vector primers M13F (CCCAGTCACGACGTTGTAAAACG) and M13R (AGCGATAACAATTTCACACAGG). For each tick, up to 5 clones with inserts of the expected length were sequenced and the rickettsial species were identified as described below.

### Amplification of *Rickettsia ompA* and *gltA*

To verify identification of unusual or uncommon *Rickettsia* spp. (such as *R. monacensis*, *R. conorii*, *R. felis* based on 23S-5S sequencing/blast search), we further screened those samples by amplifying and sequencing other gene targets, such as *ompA* and *gltA*, using respective *Rickettsia*-specific primers as shown in Additional file 1: Table S1. The PCR reactions for *gltA* and *ompA* genes were conducted in 20 μl volumes comprising 10 μl of reaction buffer, 1 μl each of forward and reverse primers (10 μM) with 1–2 μl of gDNA template in primary reactions and 1 μl of amplicons from primary PCR reaction used as the template for nested PCR reactions. The PCR conditions used for *gltA* amplification were modified from the protocol of Choi *et al*. [22] as follows: initial activation at 95°C for 10 min, followed by 30 cycles of denaturation at 94°C for 30 s, annealing at 57°C for 30 s, and extension at 72°C for 60 s, and a final extension cycle of 72°C for 5 min (for primary reaction), and 95°C for 10 min, 30 cycles of 94°C for 30 s, 55°C for 30 s, and 72°C for 60 s, and a final cycle of 72°C for 5 min (for nested reaction). The PCR conditions used for *ompA* gene were: initial activation for 10 min at 95°C, followed by 30 cycles of 95°C for 30 s, 52°C for 30 s, and 72°C for 1.30 min, and a final extension cycle of 72°C for 10 min. The same conditions were used both for primary and nested reactions. The *ompA* and *gltA* amplicons were sequenced using the forward primers of the nested PCR reactions (see Additional file 1: Table S1) as described below.

### Phylogenetic analyses of *Rickettsia* sequences

BLASTN and nucleotide sequence match analysis were used to compare partial nucleotide sequences of 23S-5S IGS clones, *ompA* and *gltA* amplicons to those in the GenBank database. Sequences were aligned with the multiple-alignment CLUSTALX software package [23]. Evolutionary distances between sequences of the 23S-5S clones, *ompA* and *gltA* amplicons and the respective genes of known *Rickettsia* species from the NCBI database were calculated (Kimura 2 parameter model) [24] and phylogenetic trees were separately constructed by the neighbor-joining method [25]. Clones from each tick that were 99-100% identical in nucleotide sequence were exhibited as one clone in constructed trees. Bootstrap analyses [26], consisting of 1,000 iterations with the MEGA 6 software package [27], were performed to evaluate the robustness of tree topologies.

### *E. chaffeensis*, *E. ewingii*, and *A. phagocytophilum*

Pathogen-specific primers and gene targets are shown in Additional file 1: Table S1 [28,29]. Tick nymphs and adults collected in 2011 were individually tested for *E. chaffeensis,* whereas those collected in 2012 were pooled (10 tick DNA samples per pool) before testing. For detection of *E. ewingii* and *A. phagocytophilum*, DNA samples were pooled (10 tick DNA samples per pool) before testing in both years to increase sample throughput because we anticipated that few ticks would be infected with these pathogens. For all three pathogens, only those tick DNA samples in positive pools were individually re-tested.

Nested PCR for *E. chaffeensis* detection targeted an antigen-expressing gene containing a variable number of tandem repeats (Additional file 1: Table S1) [3]. In the primary amplification for *E. chaffeensis*, the thermocycler was operated under the following program: denaturation at 95°C for 10 min, 40 cycles of denaturation (95°C, 30 s), annealing (55°C, 90 s) and extension (72°C, 90 s), and final extension of 72°C for 10 min. In the nested amplification, annealing and extension times were reduced to 30 and 60 s, respectively, and the cycles were repeated 30 times.

For *E. ewingii* detection, thermocycler conditions in the primary amplification were the same as for *E. chaffeensis*, except that the annealing temperature and time were changed to 60°C and 30 s, respectively, followed by extension at 72°C for 1 min. In the nested amplification, these PCR conditions were modified so that the annealing temperature was held at 61°C for 45 cycles. Unlike other pathogen detections conducted in this study, 2 μl of the primary amplification product was used in the nested PCR amplification for *E. ewingii*. Nested PCR for *A. phagocytophilum* targeted heat shock operon (*groESL*) (Additional file 1: Table S1) and was conducted under conditions previously described [30].

For *gltA* gene amplification of Panola Mountain *Ehrlichia*, the PCR conditions in the primary amplification were as follows: denaturing at 95°C, annealing at 55°C for 30 sec and an extension at 72°C for 1 min for 40 cycles followed by a final extension at 72°C for 5 min. In the nested PCR, the conditions were similar to the primary reaction except that the annealing temperature was increased to 60°C [31].

To visualize nested PCR amplicons, 5–10 μl of each PCR product was electrophoresed in a 1.2% agarose gel containing ethidium bromide in 0.5x TAE buffer.

### Sequencing *Rickettsiales*

PCR-amplified DNA from the 23S-5S *Rickettsia* clones and amplicons from nested PCR assays for *E. chaffeensis*, *E. ewingii,* and Panola Mountain *Ehrlichia*, *A. phagocytophilum*, and *Rickettsia ompA* and *gltA* were purified with QIAquick PCR purification kit or, if there were multiple-bands, bands of the proper size range were gel-extracted and purified. DNA in the purified PCR products was quantified with a NanoDrop. One primer used in the amplification was employed for sequencing *Rickettsia* spp. (M13F [23S-5S clones], 190.70p [*ompA*], RpCS896p [*gltA*]), *E. chaffeensis* (FB5C), *E. ewingii* (EWF1), *Ehrlichia* sp. (Panola Mountain) (Ehr3CS-214 F), and *A. phagocytophilum* (HS43), which was performed by Eton BioScience Inc. (Research Triangle Park, NC, USA). Bacterial species from which the DNA sequences originated were identified by comparing the nucleotide sequences with those deposited in GenBank database by means of BLASTN in NCBI [32]. The DNA sequences obtained from the screening for *E. ewingii*, which targeted the 16S ribosomal gene (Additional file 1: Table S1), were additionally analyzed with SeqMatch in the public database of 16S ribosomal gene sequences, Ribosomal Database Project (http://rdp.cme.msu.edu/seqmatch/seqmatch_intro.jsp) [33].

### GenBank accession numbers

Nucleotide sequences have been deposited in GenBank with the following accession numbers: antigen-expressing gene containing a variable number of tandem repeats (*E. chaffeensis*: KJ907737 to KJ907743); *groESL* (*E. ewingii*: KJ907744; *E. chaffeensis*: KJ907745 to KJ907753); 16S rRNA (*A. phagocytophilum*: KJ942183, KJ942185; *E. chaffeensis*: KJ942184, KJ942186, KJ942210, KJ942230, KJ942242, KJ942243; *E. ewingii*: KJ942189 to KJ942209, KJ942211, KJ942212, KJ942214 to KJ942218, KJ942222 to KJ942228, KJ942231, KJ942233, KJ942235 to KJ942237, KJ942240, KJ942241; *E. ruminantium*: KJ942213, KJ942221, KJ942239; *Rickettsia montanensis*: KJ942234; uncultured bacterium: KJ942187, KJ942188, KJ942219, KJ942220, KJ942232, KJ942238); *gltA* (Panola Mountain *Ehrlichia*: KJ796447 to KJ796449; *Rickettsia* spp.: KP172247 to KP172258); *ompA* (*Rickettsia* spp.: KP172259 to KP172268); and 23S-5S IGS (*Rickettsia* spp.: KJ796403 to KJ796446).

### Ethical statement

The study was approved by the Institutional Review Board of the University of North Carolina, Chapel Hill.

## Results

### Comparative abundance of tick species

**Figure 1:**
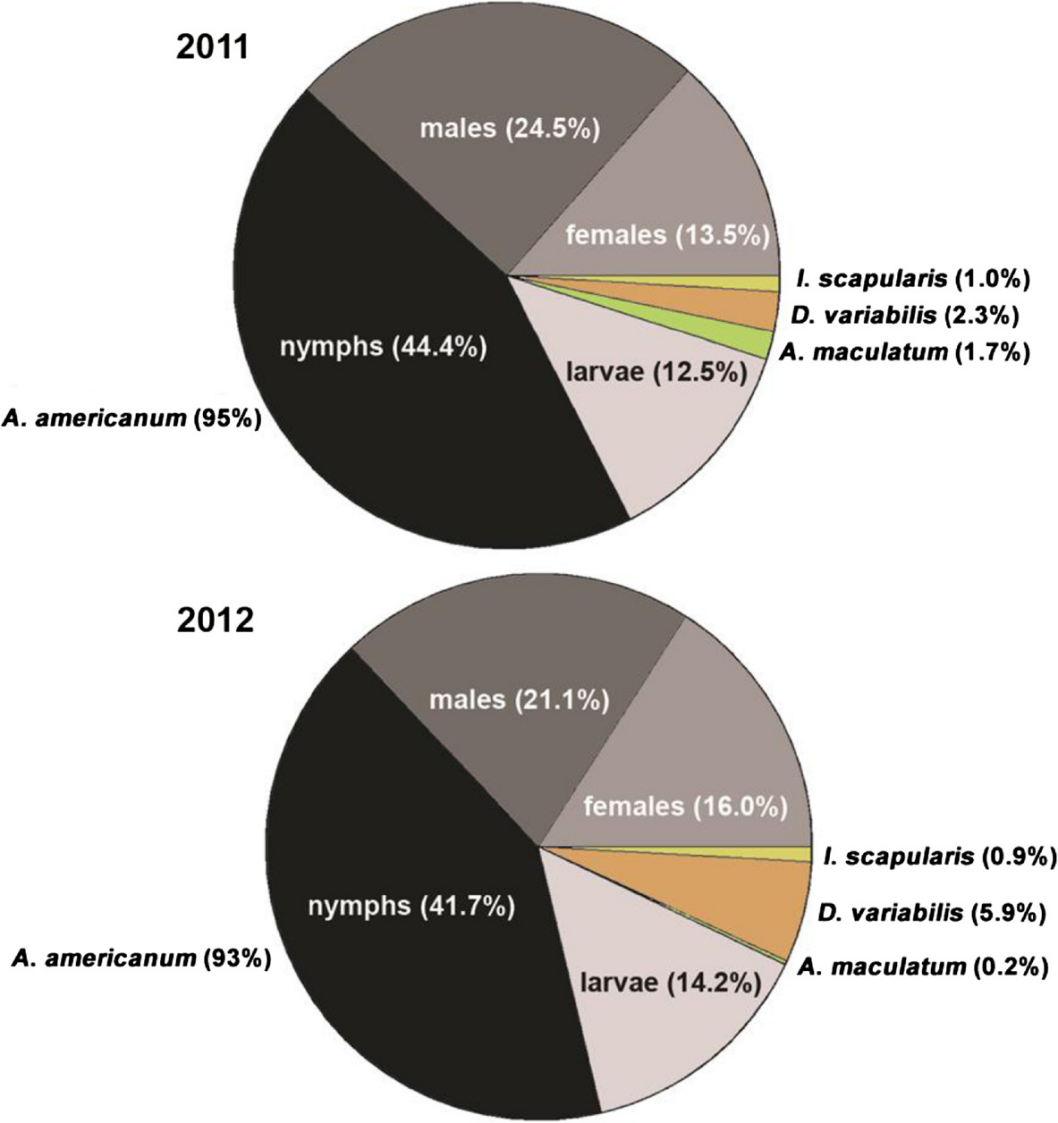
**Prevalence of tick species collected from subjects in 2011 (** ***n*** **= 423) and 2012 (** ***n*** **= 451).**

In the two-year period of this study (2011–2012), a total of 874 ticks (423 and 451 ticks in 2011 and 2012, respectively) were collected by study participants and submitted for analysis. These ticks consisted of four species; *Amblyomma americanum* (lone star tick), *Amblyomma maculatum* (Gulf Coast tick), *Dermacentor variabilis* (American dog tick) and *Ixodes scapularis* (black-legged tick) (Figure 1). The majority of ticks were identified as *A. americanum* in both years (95.0 and 92.9% in 2011 and 2012, respectively) (Figure 1). All life stages of *A. americanum* ticks were received for identification and testing, with nymphs most frequently identified, accounting for 46.8% (188/402) and 44.9% (188/419) of *A. americanum* collected in 2011 and 2012, respectively (Figure 1). The other tick species were far less frequently collected than *A. americanum* at frequencies ranging from 0.2% to 5.9% each year (Figure 1). In contrast to *A. americanum*, only adult ticks were submitted for analysis for these tick species (data not shown). Compared to 2011, *D. variabilis* increased in 2012 from 2.3% (*n* = 10) to 5.9% (*n* = 27) whereas *A. maculatum* declined from 1.7% (*n* = 7) to 0.2% (*n* = 1). The percentage of ticks identified as *I. scapularis* did not change appreciably between the two years of the study (Figure 1).

### *Rickettsia* species

Genus *Rickettsia* was the predominant putative pathogen identified. *Rickettsia* species were detected in 60.9% (479/787) of the DNA samples, including all tick species (Table 1). The infection rate was lower in 2012 (52.7%, 212/402) than in 2011 (69.4%, 267/385) and this decrease was observed across all tick species except for *D. variabilis* (Table 1). Reduction was most marked among *A. maculatum* (Table 1) and *A. americanum* larvae. Infection rates for larvae declined from 80% (12 of 15 DNA extractions) in 2011 to 13% (2 of 15 DNA extractions) in 2012.

**Table 1:** **Infection rates of** ***Rickettsiales*** **bacteria detected in nested PCR assays and identified by sequencing for tick species**

		**Number infected (% infected for each tick species)**				
**Bacteria**	**Year**	***A. americanum***	***A. maculatum***	***D. variabilis***	***I .scapularis***	**All species**
		**(** ***n*** **= 821 [734])** ^***a***^	**(** ***n*** **= 8)**	**(** ***n*** **= 37)**	**(** ***n*** **= 8)**	**(** ***n*** **= 874 [787])** ^***a***^
*Rickettsia*	2011	257 (70.6)	4 (57.1)	2 (20.0)	4 (100.0)	267 (69.4)
	2012	202 (54.6)	-^*b*^	8 (29.6)	2 (50.0)	212 (52.7)
	Total	459 (62.5)	4 (50.0)	10 (27.0)	6 (75.0)	479 (60.9)
*E. ewingii*	2011	15 (4.1)	-	-	-	15 (3.9)
	2012	37 (10.0)	-	3 (11.1)	-	40 (10.0)
	Total	52 (7.1)	-	3 (8.1)	-	55 (7.0)
*E. chaffeensis*	2011	5 (1.4)	1 (14.3)	-	-	6 (1.6)
	2012	8 (2.2)	-	-	-	8 (2.0)
	Total	13 (1.8)	1 (12.5)	-	-	14 (1.8)
*A. phagocytophilum*	2011	-	-	-	-	-
	2012	2 (0.5)	-	-	-	2 (0.5)
	Total	2 (0.3)	-	-	-	2 (0.3)

^*a*^Numbers in the brackets represent the number of DNA samples tested for *Rickettsiales* bacteria. DNA samples were extracted from individual ticks and pools of *A. americanum* larvae. In 2011, 48 larvae ticks were pooled (9 and 1 pools containing five and three larvae, respectively). In 2012, 63 ticks were pooled (11, 2 and 1 pools containing five, three and two larvae, respectively). The number of the DNA extractions was used as a denominator to calculate infection rates shown in the table.

^*b*^The minus symbol indicates that no positive specimens were found among the submitted specimens for this tick species.

**Figure 2:**
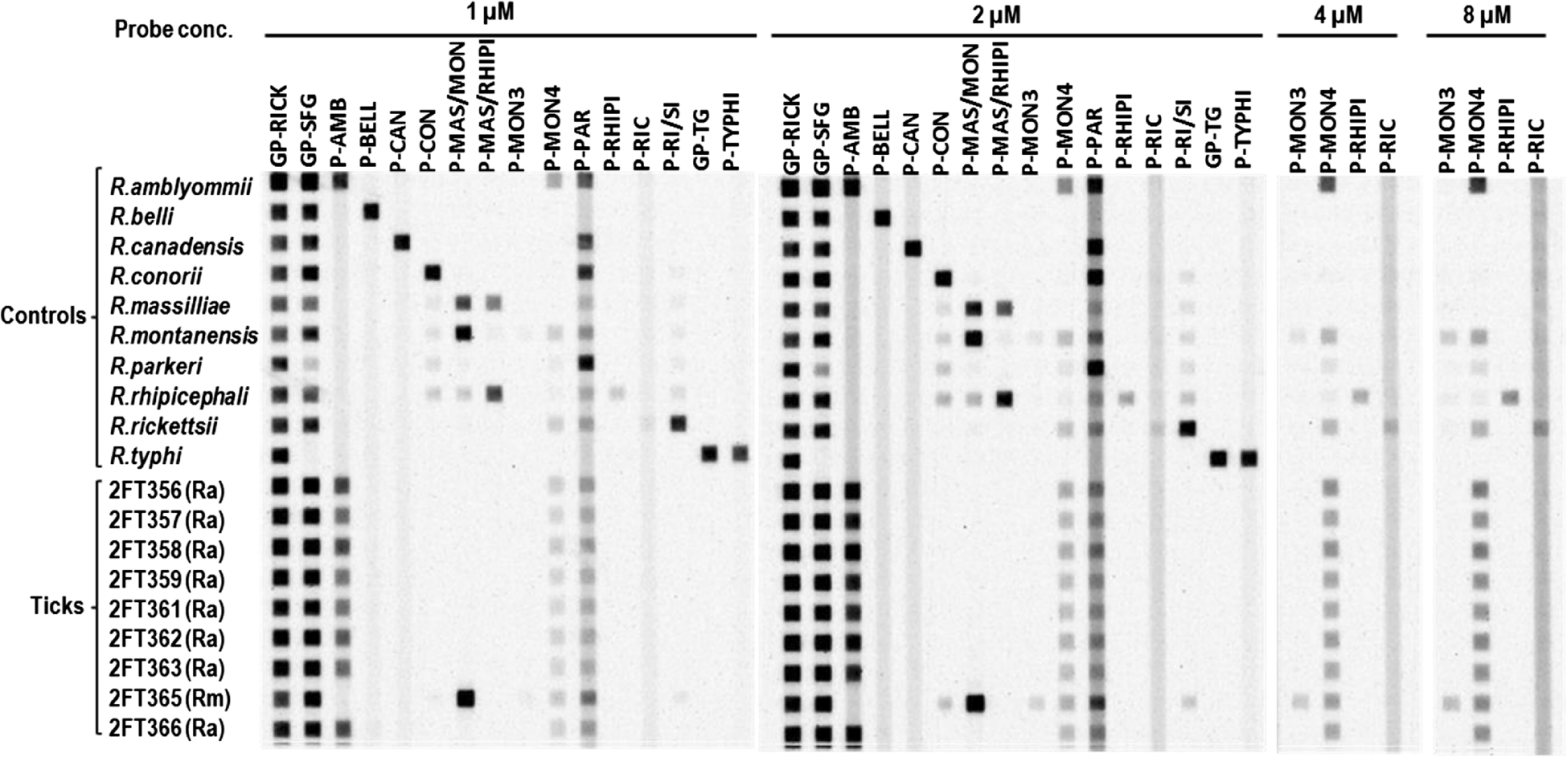
**PCR-RLB hybridization results for 23S-5S IGS fragments amplified from genomic DNA extracted from** ***Rickettsia*** **controls and** ***A*** **.** ***americanum*** **ticks.** Next to the identification code of tick samples, *Rickettsia* species identifications as determined by the RLB hybridization patterns are shown in parentheses. Ra = *R. amblyommii* and Rm = *R. montanensis*. An example of hybridization patterns for some biotin-labeled *A. americanum* 23S-5S DNAs are presented in the lower half the figure. All of the ticks exhibited strong hybridization to the *Rickettsia* genus level (GP-RICK) and SFG (GP-SFG) probes, indicating that they were infected with a spotted fever group *Rickettsia* species. All but one 23S-5S DNA samples hybridized with probe P-AMB, indicating that these ticks were infected with *R. amblyommii*. Tick DNA sample 2FT365 hybridized with probe P-MAS/MON, but failed to hybridize to P-MAS/RHIPI, indicating that the tick was infected with *R. montanensis*.

To identify the species of *Rickettsia* in 17-kDa-positive DNA samples, we developed a RLB hybridization assay. RLB hybridization patterns of 23S-5S amplicons of 10 control *Rickettsia* DNAs with four different amino labeled probe concentrations are shown in Figure 2. All control DNAs hybridized with *Rickettsia* genus probe (GP-RICK) and also hybridized with the intended DNA probes specific for SFG and TG species of *Rickettsia*.

Of 479 17-kDA-positive samples, 96.2% (461/479) of the samples were amplified with *Rickettsia*-specific 23S-5S primers. The RLB hybridization was conducted on 461 23S-5S amplicons of which 92.4% (426/461) hybridized to one or multiple probes and 7.6% (35/461) exhibited no hybridizations (unknowns). RLB hybridizations identified 24 ticks co-infected with two *Rickettsia* species, which corresponded to 3.0% (24/787) of the total and 5.2% (24/461) of the 23S-5S *Rickettsia*-positive samples. Additionally, 23S-5S amplicons of the unknowns (15 of the 35 samples), co-infected (17 of the 24 samples) and samples that were identified as *R. parkeri* or *R. montanensis* in RLB (3 samples) were successfully cloned and sequenced. “*Candidatus* R. amblyommii” was the predominant species identified in ticks, accounting for 90.2% (416/461) of the 23S-5S amplified *Rickettsia*-positive samples and 52.8% (416/787) of all of the ticks tested for pathogens (Table 2). *R. amblyommii* was detected in all four tick species, but the great majority (99%; 412/416) of *R. amblyommii*-positive ticks were *A. americanum* (Table 2). The percentage of *R. amblyommii*-infected samples among *Rickettsia* 17-kDa-positive *A. americanum* did not change appreciably between 2011 (88.7%, 228/257) and 2012 (91.1%, 184/202) (Table 2). In addition to *R. amblyommii*, RLB hybridization, sequencing and phylogenetic analyses of 23S-5S amplicons revealed that *A. americanum* harbored a diverse array of *Rickettsia* species, including *R. parkeri* and some unknown *Rickettsia* species (labelled *Rickettsia* sp. A-D in Table 2)*.* Notably *R. rickettsii* was also detected in single *A. americanum* (Table 2). These molecular isolates were 98-100% homologous to nucleotide sequences for 23S-5S IGS deposited in GenBank. Additionally, 8 amplicons from *A. americanum* and 1 amplicon from *D. variabilis* hybridized only to SFG rickettsiae probes and could not be further identified. *R. parkeri* was also detected in *A. maculatum* and *I. scapularis*, and an *R. monacensis*-like species (=*Rickettsia* sp. C) was identified in three *I. scapularis* and two *A. americanum* (Table 2).

**Table 2:** **Results of PCR-RLB hybridization assays or cloning and sequencing of 23S-5S IGS amplicons for identification of** ***Rickettsia*** **species in 17-kDa-positive ticks**

	**Year collected**	**Number (%** ^***a***^ **) of samples testing positive for each** ***Rickettsia*** **species within each tick species**				
***Rickettsia*** **species**		***A. americanum*** **[** ***n*** **= 427]** ^***b***^	***A. maculatum*** **[** ***n*** **= 4]**	***D. variabilis*** **[** ***n*** **= 4]**	***I. scapularis*** **[** ***n*** **=6]**	**All tick species** **[** ***n*** **= 441]** ^***f***^
*R. amblyommii* ^*c,d*^	2011	228 (89.8)	2 (28.6)	1 (50.0)	1 (10.0)	232 (85.0)
	2012	184 (92.5)	-^*e*^	-	-	184 (90.2)
	Total	412 (90.9)	2 (28.6)	1 (20.0)	1 (8.3)	416 (87.2)
*R. bellii* ^*c*^	2011	1 (0.4)	1 (14.3)	-	-	2 (0.7)
	2012	3 (1.5)	-	1 (33.3)	-	4 (1.9)
	Total	4 (0.9)	1 (14.3)	1 (20.0)	-	6 (1.2)
*R. massiliae* ^*c*^	2011	1 (0.4)	-	-	-	1 (0.4)
	2012	-	-	-	-	-
	Total	1 (0.2)	-	-	-	1 (0.2)
*R. montanensis* ^*c,d*^	2011	-	1 (14.3)	-	1 (10.0)	2 (0.7)
	2012	-	-	1 (33.3)	-	1 (0.5)
	Total	-	1 (14.3)	1 (20.0)	1 (8.3)	3 (0.6)
*R. parkeri* ^*c,d*^	2011	5 (2.0)	2 (28.6)	-	3 (30.0)	10 (3.7)
	2012	4 (2.0)	-	-	-	4 (2.0)
	Total	9 (2.0)	2 (28.6)	-	3 (25.0)	14 (2.9)
*R. rickettsii* ^*d*^	2011	-	-	-	-	-
	2012	1 (0.5)	-	-	-	1 (0.5)
	Total	1 (0.2)	-	-	-	1 (0.2)
*Rickettsia* sp. A^*c,d*^	2011	7 (2.8)	-	-	-	7 (2.6)
	2012	3 (1.5)	-	1 (33.3)	-	4 (2.0)
	Total	10 (2.2)	-	1 (20.0)	-	11 (2.3)
*Rickettsia* sp. B^*d*^	2011	-	-	-	-	-
	2012	2 (1.0)	-	-	-	2 (1.0)
	Total	2 (0.4)	-	-	-	2 (0.4)
*Rickettsia* sp. C^*d*^	2011	2 (0.8)	-	-	1 (10.0)	3 (1.1)
	2012	-	-	-	2 (100)	2 (1.0)
	Total	2 (0.4)	-	-	3 (27.3)	5 (1.0)
*Rickettsia* sp. D^*c*^	2011	1 (0.4)	-	-	-	1 (0.4)
	2012	-	-	-	-	-
	Total	1 (0.2)	-	-	-	1 (0.2)
Unknown *Rickettsia* ^*c*,*d,h*^	2011	3 (1.2)	1 (14.3)	-	4 (40.0)	8 (3.0)
	2012	1 (0.5)	-	-	-	1 (0.5)
	Total	4 (0.9)	1 (14.3)	-	4 (33.3)	9 (1.9)
Unknown SFG^*c*^	2011	6 (2.4)	-	1 (50.0)	-	7 (2.6)
	2012	1 (0.5)	-	-	-	1 (0.5)
	Total	7 (1.5)	-	1 (20.0)	-	8 (1.7)
Total infections	2011	254	7	2	10	273
	2012	199	0	3	2	204
	Total	453	7	5	12	477^g^

^*a*^Percentage in parenthesis was calculated using the total number of infections detected/identified for *Rickettsia* in each tick species as the denominator.

^*b*^Numbers in the brackets represent the total number of 17-kDA–positive samples that were successfully amplified and hybridized in 23S-5S PCR-RLB assay from both years of the study for each tick species.

^*c*^ *Rickettsia* species identified by reverse line blot hybridization. *R. massiliae* was identified based on hybridization to the P-MAS/MON and P-MAS/RHIPI probes but not to the P-RHIPI probe.

^*d*^ *Rickettsia* species identified by cloning and sequencing 23S-5S IGS amplicons.

^*e*^The minus symbol indicates that no positive specimens were found among the tested specimens for this tick species.

^f^Of 461 23S-5S positive samples,20 samples were not identified by RLB hybridization or through cloning and sequencing. These samples were not included in the table.

^g^Total number of infections (*n* = 477) exceeds the total number of DNA samples (*n* = 441) because some ticks were infected with more than one *Rickettsia* species.

^*h*^Includes *Rickettsia* sp. E shown in the phylogenetic tree constructed of 23S-5S IGS gene sequences (Figure 3).

23S-5S gene fragments from 35 of the 62 (co-infected and unknowns, comprising 26 *A. americanum*, 5 *I. scapularis*, 1 *D. variabilis* and 3 *A. maculatum*) samples were successfully cloned and nucleotide sequence analysis of up to 5 clones per tick revealed that 6 of the 35 ticks were co-infected. All co-infections were predominantly detected in *A. americanum*, involving *R. amblyommii* with *R. parkeri* (*n* = 3) or *Rickettsia* sp. B (*n* = 2), and *R. parkeri* with *Rickettsia* sp. A (*n* = 1). These molecular isolates were 98-100% homologous for 23S-5S IGS to nucleotide sequences deposited in GenBank.

Attempts to amplify *ompA* and *gltA* gene fragments for ticks containing *Rickettsia* sp. A that hybridized to the *R. conorii* probe were not successful. However, *Rickettsia* sp. C *ompA* and *gltA* amplicons were 96% and 99% homologous, respectively, to *R. monacensis* sequences deposited in GenBank.

### Phylogenetic analyses of *Rickettsia* nucleotide sequences

**Figure 3:**
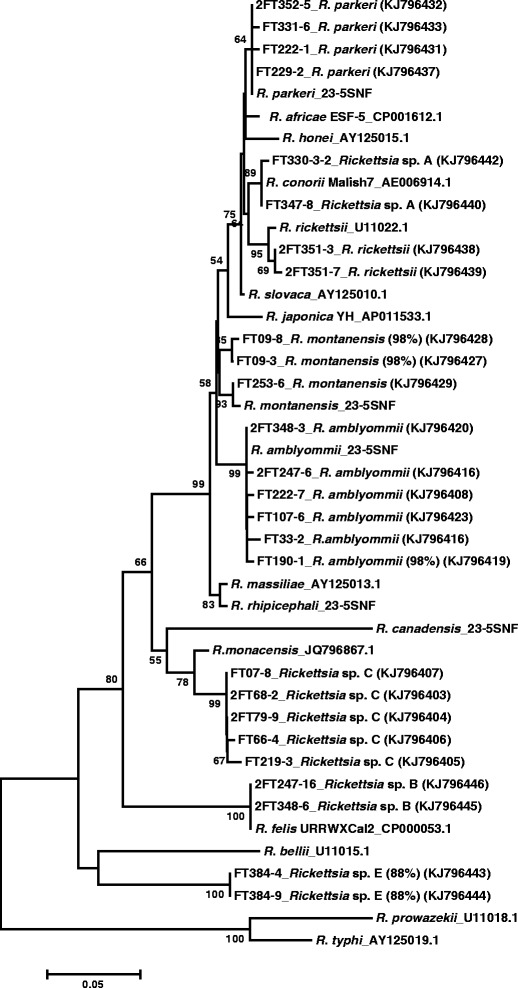
**Neighbor-joining tree showing phylogenetic relationship of partial 23S-5S IGS sequences of known** ***Rickettsia*** **and species identified after cloning of tick genomic DNA samples that were initially classified as unknown and/or were identified as co-infected samples in RLB hybridization assays.** Sequence homologies <99% are indicated in parentheses after the sequence identity. The scale bar indicates an estimated change of 5% 23S-5S IGS. Sequences beginning with “FT” or “2FT” were generated in this study. Bootstrap values below 50% are not shown in the tree branch. Accessions numbers for study samples are given in parentheses. *Unpublished GenBank sequences.

A phylogenetic tree was constructed by the neighbor-joining method to characterize the relationship between *Rickettsia* 23S-5S IGS nucleotide sequences amplified from ticks and *Rickettsia* sequences deposited in GenBank (Figure 3). Our results indicate that the ticks (especially *A. americanum*) contained a diverse array of *Rickettsia* species. The 23S-5S sequences that were 98-100% homologous to *R. amblyommii*, *R. parkeri*, *R. rickettsii,* and *R. montanensis* from BLASTN were grouped with the respective *Rickettsia* spp. from GenBank. Rickettsial species identified as *Rickettsia* sp. A and B were closely homologous to *R. conorii* and *R. felis*, respectively, in the phylogenetic tree. Sequences for *Rickettsia* sp. C that showed 98% similarity to *R. monacensis* in BLASTN were placed close to *R. monacensis* but clustered as a separate branch, suggesting the possibility of a new species or strain. Also the sequences for *Rickettsia* sp. E that had 87% similarity with *R. felis* in BLASTN search were branched independently and clustered closer to *R. bellii*, strongly supporting the presence of a novel *Rickettsia* species.

Phylogenetic trees constructed from *Rickettsia ompA* and *gltA* sequences from GenBank are shown in Additional file 2: Figures S1 and S2. *Rickettsia* spp. previously identified as *R. parkeri* and *R. amblyommii* (Figure 3) clustered with and were 99-100% homologous to their respective GenBank *ompA* and *gltA* sequences. *Rickettsia* sp. C sequences clustered close to but as a separate group from *R. monacensis*, supporting the 23S-5S phylogenetic results that showed *Rickettsia* sp. C to be a putative novel species. *Rickettsia* sp. E, detected in an *I. scapularis* female, was grouped with *Rickettsia* sp. C *ompA* sequences (Additional file 2: Figure S1) but for *gltA*, *Rickettsia* sp. E clustered near *R. cooleyi* (Additional file 2: Figure S2).

### *Ehrlichia ewingii*

*E. ewingii* was the second most frequently identified pathogen in the ticks tested (*n* = 55, 7.0%) (Table 1). This pathogen was detected in *A. americanum* (*n* = 52, 7.1%) and *D. variabilis* (*n* = 3, 8.1%) (Table 1). In 2011, 3.9% of the ticks (*n* = 15) were infected with *E. ewingii* whereas the prevalence markedly increased to 10.0% (*n* = 40) in 2012 (Tables 1). The increase in prevalence was most pronounced among *D. variabilis* (0% in 2011 vs. 11.1% in 2012) (Table 1).

In the course of screening for *E. ewingii*, sequencing of PCR amplicons revealed that some amplicons were highly homologous to non-*E. ewingii* bacteria such as *Acidobacteria* (*n* = 6), *E. chaffeensis* (*n* = 8), *E. ruminantium* (*n* = 3), *A. phagocytophilum* (*n* = 2), and *R. montanensis* (*n* = 1). These ticks were considered to be negative for *E. ewingii* and positive for the other pathogens. To confirm detection of *E. ruminantium*, a nested PCR assay targeting the citrate synthase gene (*glt*A) (Additional file 1: Table S1) was carried out for the three *A. americanum* ticks that tested positive for this agent. Sequencing of *gltA* PCR amplicons showed that these ticks were infected with an *Ehrlichia* sp. that was 99-100% homologous to the Panola Mountain *Ehrlichia*.

### *Ehrlichia chaffeensis* and *Anaplasma phagocytophilum*

A total of 1.8% of the ticks (*n* = 14) was found to be positive for *E. chaffeensis* by nested PCR assays targeting this pathogen and sequencing of amplicons obtained in the course of screening for *A. phagocytophilum* and *E. ewingii* (Table 1). The prevalence was similar in 2011 (*n* = 6, 1.6%) and 2012 (*n* = 8, 2.0%) (Table 1). A majority of the ticks infected with *E. chaffeensis* were *A. americanum* (adults and nymphs) with the exception of one adult *A. maculatum* tick collected in 2011 (Table 1).

Ticks infected with *A. phagocytophilum* were rarely detected with the infection rate of 0.3% (*n* = 2) (Tables 1). All the ticks positive for *A. phagocytophilum* were identified through sequencing amplicons attained in the course of screening *A. americanum* for *E. ewingii* in 2012 (Table 1).

### *Rickettsia-Ehrlichia* and *Rickettsia*-*Anaplasma* co-infections

Our nested PCR results revealed that 5.3% (42/787) of the ticks were positive for multiple *Rickettsiales* pathogens and such co-infections predominantly included *Rickettsia* species. Among the ticks collected in 2011, a total of 16 ticks positive for genus *Rickettsia* were also infected with *E. chaffeensis* (*n* = 5) or *E. ewingii* (*n* = 11). Two ticks containing *E. chaffeensis* contained multiple *Rickettsia* species (*R. amblyommii* + *R. bellii* and *R. amblyommii* + *R. massiliae*) based on RLB hybridization results*.* Two ticks containing *E. ewingii* contained *R. parkeri* or an unidentified *Rickettsia* species. In 2012, *A. americanum* that carried *R. amblyommii* were also found to be positive for *E. chaffeensis* (*n* = 2), *E. ewingii* (*n* = 16), or *A. phagocytophilum* (*n* = 2). Two ticks infected with *E. ewingii* and *R. amblyommii* were also infected with *Rickettsia* sp. A or *R. bellii*. 23S-5S amplicons from two of three ticks infected with *E. ewingii* and an unidentified *Rickettsia* species were cloned and sequenced. These two *A. americanum* ticks were found to contain *R. amblyommii* and either *Rickettsia* sp. B or *R. parkeri*. The only co-infection which did not involve a *Rickettsia* species was detected in one *A. americanum* female, which was collected in 2012 and infected with *E. chaffeensis* and *E. ewingii*. All co-infections were detected in *A. americanum* except for one *A. maculatum* male (collected in 2011) that carried *R. amblyommii*, *R. bellii* and *E. chaffeensis*.

## Discussion

### Comparative abundance of tick species

Ticks collected by NC foresters, park service, and wildlife personnel from their skin were predominantly *A. americanum* with small numbers of *D. variabilis*, *I. scapularis* and *A. maculatum*. All four species have been reported to parasitize humans; however, within its geographic range, *A. americanum* generally dominated collections [34-38]. In northeastern states, *I. scapularis* was the most frequently collected tick [39], but this tick and *A. maculatum* have been infrequently collected from humans in southern states [35,38,40].

### *Rickettsia* identified in ticks removed from the skin of outdoor workers

Few comparable studies of the *Rickettsiales* pathogens contained in North American ticks removed from humans have been conducted. Our results indicate that outdoor workers in NC would experience the greatest risk of receiving ticks bites from *A. americanum*. Based on past studies [16,18], we expected that *R. amblyommii* would be prevalent in the *A. americanum* tested. PCR-RLB hybridization provided a high throughput method for screening ticks for this and other *Rickettsia* species. In our investigation, *R. amblyommii* accounted for 86.8% (416/479) of the *Rickettsia* species in 17-kDa-positive ticks removed from the skin of outdoor workers. Likewise, in past studies, *R. amblyommii* was the most frequently detected SFG *Rickettsia* in *A. americanum* and *D. variabilis* removed from the skin of people [38,41,42]. Similarly in other recent investigations of host-seeking ticks, *R. amblyommii* was the most prevalent species of *Rickettsia* detected in *A. americanum* [16,18,43-48].

Because RLB hybridization probes used in our assay were 17–25 bp in size, we anticipated some heterologous hybridizations would occur between ~400 bp 23S-5S amplicons and RLB probes. Accordingly, we cloned and sequenced 23S-5S gene fragments for some ticks containing *Rickettsia* species that failed to hybridize to any genus, group or species-specific probes or that gave unexpected hybridizations for particular tick species. RLB hybridization indicated that some ticks were co-infected but cloning and sequencing results indicated that only one tick contained two *Rickettsia* species. However, since only up to 5 clones per tick (1–2 clones for some ticks) were sequenced, it is likely that sequencing additional clones would have detected uncommon *Rickettsia* species. Albeit, cloning and sequencing of co-infected ticks identified *Rickettsia* species that were unexpected. *Rickettsia* species C, that was 98% homologous to *R. monacensis* for 23S-5S sequences, was detected in two *A. americanum* and three *I. scapularis* ticks. To our knowledge this *Rickettsia* species has not been detected in field-collected *A. americanum* or *I. scapularis*, but *R. monacensis* has been detected in *I. ricinus* in Europe [49,50]. However, our phylogenetic analyses showed that 23S-5S clone sequences clustered near GenBank *R. monacensis* sequences but on a separate branch, suggesting that the *Rickettsia* species might be a close relative of *R. monacensis*. Subsequent phylogenetic analyses of *Rickettsia ompA* and *gltA* sequences support this conclusion. Partial nucleotide sequences from several *A. americanum* clones clustered closely with *R. conorii* and *R. rickettsii. R. conorii* is the agent causing Mediterranean spotted fever and a variety of other rickettsioses in areas of Europe and Asia [51], so it is likely that the rickettsial species (*Rickettsia* sp*.* A) that we detected is a variant of *R. conorii. R. rickettsii*, the causal agent of Rocky Mountain spotted fever, has been identified previously in *A. americanum* [44,52]. Sequence matches for *R. parkeri* were obtained for clones from 6 *A. americanum* and one *A. maculatum. R. parkeri* has been detected previously in *A. americanum* from Georgia and Tennessee [53] and Virginia [45]. This tick has been shown to be a competent laboratory vector for this rickettsial species [54]. *R. parkeri* is an established human pathogen [55] that is vectored by *A. maculatum* [56].

In addition to *Rickettsia* sp. A and C, ticks contained other novel *Rickettsia* spp. Classification of *Rickettsia* sp. E as a putative novel species is supported by phylogenetic analyses of 23S-5S, *ompA* and *gltA* sequences. *Rickettsia* spp. B and D that are closely related to *R. felis* and *R. typhi*, respectively, are likely to be new species as well. Similarly, Heise *et al*. [43] detected novel *Rickettsia* sp. in *A. americanum* by amplifying, cloning and sequencing 17 kDa and *gltA* genes.

### Other *Rickettsiales* organisms detected in ticks

*E. chaffeensis* and *E. ewingii* are pathogens vectored by *A. americanum* [57] that cause human illness [58]. Stromdahl *et al*. [59] tested ticks removed from military personnel by PCR for common bacterial pathogens and reported finding 15% of *A. americanum* ticks containing a 16S rRNA amplicon that matched the expected size of *E. chaffeensis*. Both pathogens have been reported in recently published molecular surveys of ticks [16-18,60-63]. Mean infection rates were geographically variable but generally similar to the rates that we observed (1.8% for *E. chaffeensis and* 7.1% for *E. ewingii*). In previous studies, infection rates for *E. chaffeensis* ranged from a low of 2.0% for *A. americanum* in Georgia [61] to a high of 6.7% in Missouri [60]. In comparison, *E. ewingii* infection rates ranged from 0.8% for ticks in Tennessee [63] to 6.0% for Mississippi [62].

An *Ehrlichia* species 99% homologous (based on the 16S rRNA gene target) to *E. ruminantium* was detected in *A. americanum*. Subsequent sequence analysis of *gltA* gene fragments established that the tick was infected with the Panola Mountain *Ehrlichia*, a variant of *E. ruminantium* [31]. The Panola Mountain *Ehrlichia* is pathogenic to some domestic animals [64,65] and humans [66]. This *Ehrlichia* species has not been reported to occur in *A. americanum* in North Carolina but has been detected in ticks collected in 3 other southern states [31].

## Conclusions

In the southern U.S., illness from spotted fever rickettsioses, including Rocky Mountain spotted fever, has escalated markedly over the past decade while case fatalities have declined [67]. Concurrently, recent studies [44,46,68,69] have failed to detect *R. rickettsii* in *D. variabilis*, an established vector of RMSF, which could account for the increased morbidity and decreased case fatality. Our results indicate that host-seeking *A. americanum* are infected with novel *Rickettsia* species. Further investigations are warranted to determine if these *Rickettsia* cause human illness. As suggested previously by Apperson *et al*. [17], these studies “…would include cell culture and molecular evaluation of human specimens from clinically ill patients to provide specific identity of the etiologic agent”.

## Additional files

Additional file 1: Table S1. Nucleotide sequences of primers used in nested PCR. **Table S2.** Nucleotide sequences of primers and probes used in the PCR-RLB hybridization assay. Additional file 2: Figure S1. Neighbor-joining phylogenetic analysis of partial *ompA* gene sequences (488 bp), showing the relationships between known *Rickettsia* spp. and sequences that were PCR amplified from *A. americanum* and *I. scapularis* ticks. The scale bar indicates an estimated change of 2% in *ompA* (outer membrane protein **A)** sequences. Sequences beginning with “FT” or “2FT” were generated in this study. Accession numbers are added in parenthesis. Bootstrap values (>50 %) based on 1000 iterations are shown at branch nodes. **Figure S2.** Neighbor-joining phylogenetic analysis of partial *gltA* (citrate synthase) gene sequences (299 bp), showing the relationships between known *Rickettsia* spp. and sequences that were PCR amplified from *A. americanum*, *A. maculatum* and *I. scapularis* ticks. The scale bar indicates an estimated change of 1% in *gltA* sequences. Sequences beginning with “FT” or “2FT” were generated in this study. Accession numbers are added in parenthesis. Bootstrap values (>50 %) based on 1000 iterations are shown at branch nodes.

## Abbreviations

SFR: Spotted Fever Rickettsioses
SFG: Spotted Fever Group
TG: Typhus Group
RMSF: Rocky Mountain spotted fever
RLB: Reverse-Line Blot
