# Supplementary material for: Prevalence of Rickettsiales in ticks removed from the skin of outdoor workers in North Carolina
Source: Parasit Vectors. 2014 Dec 23;7:607. doi: 10.1186/s13071-014-0607-2 (PMC4301950; doi:10.1186/s13071-014-0607-2)
Supplement: Additional file 1: Table S1. — Nucleotide sequences of primers used in nested PCR. Table S2. Nucleotide sequences of primers and probes used in the PCR-RLB hybridization assay. [file 13071_2014_607_MOESM1_ESM.docx]

Submitted for publication in:

***Parasites and Vectors***

Prevalence of *Rickettsiales* in Ticks Removed from the Skin of Outdoor Workers in North Carolina

**Authors:** Sangmi Lee^1*^, Madhavi Kakumanu^1^, Loganathan Ponnusamy^1^, Meagan Vaughn^2^, Sheana Funkhouser^2^, Haley Thornton^1^, Steven R. Meshnick^2^ and Charles S. Apperson^1#^

^1^Department of Entomology, North Carolina State University, Raleigh, NC, 27695

^2^Department of Epidemiology, Gillings School of Global Public Health, University of North Carolina, Chapel Hill, NC, 27599

*Present address: National Institutes of Health, Bethesda, MD, 20892

^#^Corresponding author. Mailing address: Department of Entomology, Campus Box 7647, North Carolina State University, Raleigh, NC 27695-7647. Phone: (919) 515-4326. Fax: (919) 515-3748. E-mail: apperson@ncsu.edu.

# Additional file 1: Table S1 Nucleotide sequences of primers used in nested PCR.

| Organism (reference) | Primers | Sequence (5' to 3') | Amplification | Target gene (nested/semi-nested amplification product size) |
| --- | --- | --- | --- | --- |
| *Rickettsia* (1) | 17kD1_F | GCTCTTGCAACTTCTATGTT | Primary | 17-kDa protein gene  (232 bp) |
|  | 17kD2_R | CATTGTTCGTCAGGTTGGCG | Primary |  |
|  | 17kN1_F | CATTACTTGGTTCTCAATTCGGT | Nested |  |
|  | 17kN2_R | GTTTTATTAGTGGTTACGTAA | Nested |  |
| *Rickettsia* (2, 3) | 190.70p | ATGGCGAATATTTCTCCAAAA | Primary | *ompA*  (532 bp) |
|  | 190.701n | GTTCCGTTAATGGCAGCATCT | Primary |  |
|  | 190.70p | ATGGCGAATATTTCTCCAAAA | Semi-nested |  |
|  | 190.602n | AGTGCAGCATTCGCTCCCCCT | Semi-nested |  |
| *Rickettsia* (3, 4) | RpCS.877p | GGGGGCCTGCTCACGGCGG | Primary | *gltA* (338 bp) |
|  | RpCS1258n | ATTGCAAAAAGTACAGTGAACA | Primary |  |
|  | RpCS896p | GGCTAATGAAGCAGTGATAA | Nested |  |
|  | RpCS1233n | GCGACGGTATACCCATAGC | Nested |  |
| *Ehrlichia* sp. (Panola | Ehr3CS-185F | GCCACCGCAGATAGTTAGGGA | Primary | *gltA* gene  (400 bp) |
| Mountain) (5) | Ehr3CS-777R | TTCGTGCTCGTGGATCATAGTTTT | Primary |  |
|  | Ehr3CS-214F | CATTTCCACAGCATTCTCATC | Nested |  |
|  | Ehr3CS-619R | TGAGCTGGTCCCCACAAAGTT | Nested |  |
| *E. chaffeensis* (6) | FB5A | GTGACATCTTAGTTTAATAGAAC | Primary | Antigen expressing gene containing a variable number of tandem repeats  (391-841 bp) |
|  | FB3A | AAGACTGAAACGTTATAGAG | Primary |  |
|  | FB5C | GTTGATCATGTACCTGTGTG | Nested |  |
|  | FB3 | GCCTAATTCAGATAAACTAAC | Nested |  |
| *E. ewingii* (7) | EC12A | TGATCCTGGCTCAGAACGAACG | Primary | 16S rRNA gene  (403 bp) |
|  | EC11I | GGACTACIAGGGTATCTAAT | Primary |  |
|  | EWF1 | TCGAACGAACAATTCCTAAA | Nested |  |
|  | HE3 | TATAGGTACCGTCATTATCTTCCCTAT | Nested |  |
| *A. phagocytophilum* (8) | HS1a | AITGGGCTGGTAITGAAAT | Primary | Heat shock operon (*groESL*) (1297 bp) |
|  | HS6a | CCICCIGGIACIAIACCTTC | Primary |  |
|  | HS43 | AT[A/T]GC[A/T]AA[G/A]GAAGCATAGTC | Nested |  |
|  | HSVR | CTCAACAGCAGCTCTAGTAGC | Nested |  |

**Table** S2 Nucleotide sequences of primers and probes used in the PCR-RLB hybridization assay

| Organism(s)*^a^* | Primers | Probes | Sequence (5' to 3') | Reference |
| --- | --- | --- | --- | --- |
| *Rickettsia* | RCK/23-5-F |  | GATAGGTCRGRTGTGGAAGCAC | 9 |
|  | RCK/23-5-R |  | TCGGGAYGGGATCGTGTGTTTC | 9 |
| *Rickettsia* |  | GP-RICK | TAGCTCGATTGRTTTACTTTG | 9 |
| SFG (Spotted Fever Group) |  | GP-SFG | ACTCACAARGTTATCAGGT | 9 |
| *R. amblyommii* |  | P-AMB | GATATTAAGAACTTTTGTGAGTTG | This study |
| *R. bellii* |  | P-BELL | GTGTTTATTCTATAATATGTCAG | 9 |
| *R. canadensis* |  | P-CAN | CMACAATATTTTCTATCTTTTG | This study |
| *R. conorii* |  | P-CON | GTTATATACTGTAGCCCTG | 9 |
| *R. massiliae,* *R. montanensis* |  | P-MAS/MON | ATATGATGTTAAGAATTTTTT | This study |
| *R. massiliae,* *R. rhipicephali* |  | P-MAS/RHIPI | TTATTGCATCACTAATGTTA | This study |
| *R. montanensis* |  | P-MON3 | TTACATATGTATATAGTGT | This study |
| *R. amblyommii*, *R. montanensis,* *R. rickettsii* |  | P-MON4 | GATATCCAGCGAAAATTATT | This study |
| *R. canadensis*, *R. conorii,* *R. parkeri* |  | P-PAR | GCTGAGTATCGTATAAAAGTTA | This study |
| *R. rhipicephali* |  | P-RHIPI | GTTAAGATATAATGTTAAG | This study |
| *R. rickettsii*, *R*. *sibirica* |  | P-RI/SI | GTTATACTGTAGTCCTGCAA | 9 |
| *R. rickettsii* |  | P-RIC | CATTAACAGTAGACTATAATAC | This study |
| TG (Typhus Group) |  | GP-TG | GTTATTCTATCGTTTTATGTYACG | 9 |
| *R. typhi* |  | P-TYPHI | ATGTCACGATTTGACCGTAAGATC | 9 |

^a^Specificity was determined based on hybridization with control DNAs representing 10 *Rickettsia* species.

**REFERENCES**

1. Oliveira KA, Oliveira LS, Dias CC, Silva, Jr A, Almeida MR, Almada G, Bouyer DH, Galvao MA, Mafra, C: **Molecular identification of *Rickettsia felis* in ticks and fleas from an endemic area for Brazilian Spotted Fever.** *Mem Inst Oswaldo Cruz* 2008, **103:**191–194.
2. Roux V, Fournier PE, Raoult D: **Differentiation of spotted fever group rickettsiae by sequencing and analysis of restriction fragment length polymorphism of PCR-amplified DNA of the gene encoding the protein rOmpA.** *J Clin Microbiol* 1996, **34**:2058–2065.
3. Regnery RL, Spruill CL, Plikaytis BD: **Genotypic identification of rickettsiae and estimation of intraspecies sequence divergence for portions of two rickettsial genes.**  *J* *Bacteriol* 1991, **173**:1576–1589.
4. Choi YJ, Jang EJ, Kim JH, Ryu JS, Lee SH, Park KH, Paik HS, Koh YS, Choi MS, Kim IS: [**Spotted fever group and typhus group rickettsioses in humans, South Korea**](http://wwwnc.cdc.gov/eid/article/11/2/04-0603.htm)**.** *Emerg Infec Dis* 2005, **11**: 237–244.
5. Loftis AD, Mixson TR, Stromdahl EY, Yabsley MJ, Garrison LE, Williamson PC, Fitak RR, Fuerst PA, Kelly DJ, Blount KW: **Geographic distribution and genetic diversity of the *Ehrlichia* sp. from *Panola Mountain* in *Amblyomma americanum*.** *BMC Infect Dis* 2008, **8:**54.
6. Sumner JW, Childs JE, Paddock CD: **Molecular cloning and characterization of the *Ehrlichia* *chaffeensis* variable-length PCR target: an antigen-expressing gene that exhibits interstrain variation.** *J Clin Microbiol* 1999, **37:**1447–1453.
7. Buller RS, Arens M, Hmiel SP, Paddock CD, Sumner JW, Rikhisa Y, Unver A, Gaudreault-Keener M, Manian FA, Liddell AM, Schmulewitz N, Storch GA: ***Ehrlichia* *ewingii*, a newly recognized agent of human ehrlichiosis.** *N Engl J Med* 1999, **341:**148–155.
8. Nicholson WL, Castro MB, Kramer VL, Sumner JW, Childs JE: **Dusky-footed wood rats (*Neotoma fuscipes*) as reservoirs of granulocytic Ehrlichiae (Rickettsiales: Ehrlichieae) in northern California.** *J Clin Microbiol* 1999, **37:**3323–3327.
9. Jado I, Escudero R, Gil H, Jimenez-Alonso MI, Sousa R, Garcia-Perez AL, Rodriguez-Vargas,M, Lobo B, Anda P: **Molecular method for identification of *Rickettsia* species in clinical and environmental samples.** *J Clin Microbiol* 2006, **44:**4572–4576.
