# Supplementary material for: Prevalence of Rickettsiales in ticks removed from the skin of outdoor workers in North Carolina
Source: Parasit Vectors. 2014 Dec 23;7:607. doi: 10.1186/s13071-014-0607-2 (PMC4301950; doi:10.1186/s13071-014-0607-2)
Supplement: Additional file 2: Figure S1. — Neighbor-joining phylogenetic analysis of partial ompA gene sequences (488 bp), showing the relationships between known Rickettsia spp. and sequences that were PCR amplified from A. americanum and I. scapularis ticks. The scale bar indicates an estimated change of 2% in ompA (outer membrane protein A) sequences. Sequences beginning with “FT” or “2FT” were generated in this study. Accession numbers are added in parenthesis. Bootstrap values (>50 %) based on 1000 iterations are shown at branch nodes. Figure S2. Neighbor-joining phylogenetic analysis of partial gltA (citrate synthase) gene sequences (299 bp), showing the relationships between known Rickettsia spp. and sequences that were PCR amplified from A. americanum, A. maculatum and I. scapularis ticks. The scale bar indicates an estimated change of 1% in gltA sequences. Sequences beginning with “FT” or “2FT” were generated in this study. Accession numbers are added in parenthesis. Bootstrap values (>50 %) based on 1000 iterations are shown at branch nodes. [file 13071_2014_607_MOESM2_ESM.docx]

Submitted for publication in:

***Parasites and Vectors***

Prevalence of *Rickettsiales* in Ticks Removed from the Skin of Outdoor Workers in North Carolina

**Authors:** Sangmi Lee^1*^, Madhavi Kakumanu^1^, Loganathan Ponnusamy^1^, Meagan Vaughn^2^, Sheana Funkhouser^2^, Haley Thornton^1^, Steven R. Meshnick^2^ and Charles S. Apperson^1#^

^1^Department of Entomology, North Carolina State University, Raleigh, NC, 27695

^2^Department of Epidemiology, Gillings School of Global Public Health, University of North Carolina, Chapel Hill, NC, 27599

*Present address: National Institutes of Health, Bethesda, MD, 20892

^#^Corresponding author. Mailing address: Department of Entomology, Campus Box 7647, North Carolina State University, Raleigh, NC 27695-7647. Phone: (919) 515-4326. Fax: (919) 515-3748. E-mail: apperson@ncsu.edu.

**Figure Captions**

**Figure 1** Neighbor-joining phylogenetic analysis of partial *ompA* gene sequences (488 bp), showing the relationships between known *Rickettsia* spp. and sequences that were PCR amplified from *A*. *americanum* and *I*. *scapularis* ticks. The scale bar indicates an estimated change of 2% in *ompA* (outer membrane protein A) sequences. Sequences beginning with “FT” or “2FT” were generated in this study. Accession numbers are added in parenthesis. Bootstrap values (>50 %) based on 1000 iterations are shown at branch nodes.

**Figure 2** Neighbor-joining phylogenetic analysis of partial *gltA* (citrate synthase) gene sequences (299 bp), showing the relationships between known *Rickettsia* spp. and sequences that were PCR amplified from *A*. *americanum* and *I*. *scapularis* ticks. The scale bar indicates an estimated change of 1% in *gltA* sequences. Sequences beginning with “FT” or “2FT” were generated in this study. Accession numbers are added in parenthesis. Bootstrap values (>50 %) based on 1000 iterations are shown at branch nodes.

***R. sibrica* (U43807.1)**

***R. mongolotimonae* (U43796.1)**

***Rickettsia* sp. S (U43805.1)**

***R. africae* (U43790.1)**

***R. parkeri* (U43802.1)**

**FT331_*R*. *parkeri* (KP172268)**

***Rickettsia* sp. Thai typhus (U43809.1)**

***R. slovaca* (U43808.1)**

***R*. *rickettsii* (U43804.1)**

***R. conorii* Isreali typhus (U43797.1)**

***R. conorii* Malish (U43806.1)**

***R. japonica* (U43795.1)**

***R. aeschilimannii* MC16 (U43800.1)**

***R. rhipicephali* (U43803.1)**

***R. massiliae* (U43799.1)**

***Rickettsia* sp. Bar (U43792.1)**

**FT222_*R. amblyommii* (KP172265)**

**2FT348_*R. amblyommii* (KP172267)**

**2FT351_ *R. amblyommii* (KP172266)**

***R. amblyommii* (JX867426.1)**

**FT330_*R. amblyommii* (KP172264)**

***R. montanensis* (U43801.1)**

***R. monacensis* (AF201329.1)**

**FT07_*Rickettsia* sp. C (KP172263)**

**FT66_*Rickettsia* sp. C (KP172259)**

**2FT68_*Rickettsia* sp. C (KP172262)**

**2FT79_*Rickettsia* sp. C (KP172261)**

**FT384_*Rickettsia* sp. C (KP172260)**

**99**

**100**

**89**

**97**

**99**

**98**

**98**

**54**

**99**

**100**

**95**

**97**

**56**

**55**

**59**

**62**

**0.02**

Fig. 1

***R. mongolotimonae* (U59731.1)**

***R. conorii* Israeli typhus (U59727.1)**

***R. sibrica* (U59734.1)**

***R. parkeri* (U59732.1)**

***Rickettsia* sp. S (U59735.1)**

***R. africae* (U59733.1)**

***Rickettsia* sp. Thai typhus (U59726.1)**

**FT331-6_*R. parkeri* (KP172256)**

***R. rickettsii* (U59729.1)**

***R. conorii* Malish (U59730.1)**

***R. slovaca* (U59725.1)**

**2FT348_*R. amblyommii* (KP172252)**

**FT114_*R. amblyommii* (KP172253)**

**FT222_*R. amblyommii* (KP172254)**

**FT229_*R. amblyommii* (KP172255)**

***R. amblyommii* (AY375163.1)**

**2FT351_*R. amblyommii* (KP172257)**

***R. japonica* (U59724.10)**

***R. aeschlimannii* MC16 (U59722.1)**

***R. rhipicephali* (U59721.1)**

***Rickettsia* sp Bar (U59720.1)**

***R. massiliae* (U59719.1)**

***R. montanensis* (U74756.1)**

**2FT247_*R. montanensis* (KP172258)**

***R. typhi* (U59714.1)**

***R. australis* (U59718.1)**

***R. helvetica* (U59723.1)**

***R. canadensis* (U59713.1)**

***R. felis* (EU853837.1)**

***R. monacensis* (AY048817.1)**

**FT07_*Rickettsia* sp. C (KP172247)**

**FT66_*Rickettsia* sp. C (KP172248)**

**2FT68_*Rickettsia* sp. C (KP172249)**

**2FT79_*Rickettsia* sp. C (KP172250)**

***R. cooleyi* (AF031536.1)**

**FT384_*Rickettsia* sp. C (KP172251)**

***R. belli* (U59716.1)**

**98**

**66**

**91**

**60**

**75**

**86**

**57**

**62**

**89**

**0.01**

Fig. 2
